# Fabrication of Progesterone-Loaded Nanofibers for the Drug Delivery Applications in Bovine

**DOI:** 10.1186/s11671-016-1781-2

**Published:** 2017-02-14

**Authors:** Chitra Karuppannan, Mehnath Sivaraj, J. Ganesh Kumar, Rangasamy Seerangan, S. Balasubramanian, Dhinakar Raj Gopal

**Affiliations:** 1 Translational Research Platform for Veterinary Biologicals, Chennai, India; 2 Tamil Nadu Veterinary and Animal Sciences University, Chennai, India

**Keywords:** Zein, Electrospinning, Nanofibers, Progesterone, Estrus synchronization

## Abstract

Progesterone is a potent drug for synchronization of the estrus and ovulation cycles in bovine. At present, the estrus cycle of bovine is controlled by the insertion of progesterone-embedded silicone bands. The disadvantage of nondegradable polymer inserts is to require for disposal of these bands after their use. The study currently focuses on preparation of biodegradable progesterone-incorporated nanofiber for estrus synchronization. Three different concentrations (1.2, 1.9, and 2.5 g) of progesterone-impregnated nanofibers were fabricated using electrospinning. The spun membrane were characterized by scanning electron microscopy, X-ray diffraction, differential scanning calorimetry, thermogravimetric analysis, and Fourier transform infrared spectroscopy. Uniform surface morphology, narrow size distribution, and interaction between progesterone and zein were confirmed by SEM. FTIR spectroscopy indicated miscibility and interaction between zein and progesterone. X-ray analysis indicated that the size of zein crystallites increased with progesterone content in nanofibers. Significant differences in thermal behavior of progesterone-impregnated nanofiber were observed by DSC. Cell viability studies of progesterone-loaded nanofiber were examined using MTT assay. In vitro release experiment is to identify the suitable progesterone concentration for estrus synchronization. This study confirms that progesterone-impregnated nanofibers are an ideal vehicle for progesterone delivery for estrus synchronization of bovines.

## Background

Electrospinning is a technique used to form nanoscale fibers. It is quite versatile for fabricating nanofibers from various synthetic or natural polymers [1]. In literature [2], reported functional electrospun nanofibrous composite structures can also be produced by incorporating functional additives in the fiber matrix or on the fiber surface. The development of nanostructured systems for the delivery and sustained release of molecules towards specific targets represents a frontier area of nanoscience and nanotechnology, with the possibility of contributing substantially to advances in animal reproduction [3]. Improving delivery techniques that minimize toxicity of drug has a significant effect on its efficacy. Overall, nanosized delivery systems enhance the therapeutic efficacy of several bioactive molecules, including reproductive hormones, by simply improving their pharmacokinetic and pharmacodynamic properties [4]. These systems are able to carry a wide variety of molecules enhancing their sustained release, showing low systemic toxicity, allowing targeted treatment, and avoiding premature inactivation [5, 6]. Electrospun polymer-based fibers have been investigated for providing different types of controlled drug release profiles, such as immediate, delayed, sustained, and biphasic releases [7, 8]. Among them, sustained drug release is gaining considerable attention as a method of administering and maintaining desired drug concentrations in the blood within a specified therapeutic window [9–11].

Zein is a mixture of proteins with different molecular weights in corn gluten. Apart from biodegradability and biocompatibility, zein has low hydrophilicity, high elasticity, and film-forming capabilities, and it is considered a potential raw material for bioengineering application [12].

The artificial induction and synchronization of estrus in production animals is critical to ensure a positive balance of the cost-benefit equation of the artificial insemination related activities. The usual administration of hormones must be very precise. The controlled hormone release is a current technological challenge. One interesting agent to be tested in such delivery system is the progesterone, a steroid hormone naturally produced by the corpus luteum of the ovaries of mammals and involved in their pregnancy. In veterinary medicine, exogenous progesterone is used as a potent drug for suppression of estrus and ovulation, making possible the synchronization of the estrus and ovulation cycles in livestock animals [13]. In this sense, the present study aims to investigate the release characteristics of progesterone-impregnated zein nanofiber obtained by electrospinning process. In addition, the ability of progesterone-loaded zein nanofibers to provide sustained drug release was studied.

## Methods

### Materials

Zein from corn and progesterone were purchased from Sigma-Aldrich (USA). Ethanol 99.7% purity was supplied by Merck

### Progesterone-Loaded Nanofiber Fabrication

Zein was dissolved in ethanol and kept under vigorous stirring overnight at room temperature. Various concentrations (1.2, 1.9, and 2.5 g) of progesterone were dissolved in ethanol for an hour at room temperature. Both solutions were mixed for an hour. Progesterone-loaded zein fibers prepared by electrospinning were spun using a voltage of 24 kV, working distance of 12 cm, and feed rate of 2 μL min^−1^. Electrospinning processes were carried out under ambient conditions (24 ± 3 °C with relative humidity 57 ± 4%) [14].

### Characterization of Progesterone Loaded Zein Nanofiber

#### Scanning Electron Microscopy

Scanning electron microscopy is used to check the surface morphology of three different concentrations (1.2, 1.9, and 2.5 g) of progesterone-incorporated nanofiber. The SEM characterization of electrospun nanofiber was performed using JEOL JSM-6480 V (accelerative voltage 20 kV) scanning electron microscopy at the Nanotechnology Department of SRM University, Chennai. The nanofiber samples collected on the aluminum foil was peeled out and then mounted on SEM sample holder using graphite-impregnated adhesive conductive black carbon tape, coated with platinum, and visualized under SEM at various magnifications.

#### X-ray Diffraction

XRD patterns were generated from nonwoven fibrous mat using a Rigaku D/Max ULTIMA 11 X-ray diffractometer (Japan). The X-rays are generated by a cathode ray tube filtered to produce monochromatic radiation directed towards the sample. The interaction of the incident rays with the sample produces constructive interference (and diffracted rays). The diffracted intensity were recorded from 0 to 1400 at 2θ angle and the pattern was recorded by Cu K radiation with 1.5418 Å and graphite monochromatic filtering wave at a tube voltage of 40 kV and tube current of 30 mA, and scanning in the region of 0 to 70 at 6 min^−1^ with incident beam.

#### Differential Scanning Calorimetry

Differential scanning calorimetry (DSC) measurements (Mettler Toledo DSC 821e, Schwerzenbach, Switzerland) were performed on samples of 5 mg in the range of −100 to 200 °C at a heating rate of 10 °C/min (N_2_ atmosphere 80 L/min). The glass transition temperature (Tg) was evaluated with the Stare-software version 6.01 (Mettler Toledo, Schwerzenbach, Switzerland; calibration with indium and zinc). Zein films were stored over silica gel or at different relative humidities for 5 days prior to measurement to achieve different water contents. The relative humidity (r.h.) was controlled by saturated salt solutions (KCH_3C_OO 22% r.h.; NaCl 75% r.h.; ZnSO_4_ 85% r.h.; pure water 100% r.h.) The predicted Tg values were calculated with the Gordon-Taylor-equation.

#### Fourier Transform Infrared Spectroscopy

Nanofiber functional groups were analyzed using FTIR spectroscopy. A pinch of the sample was placed into the sample holder and FT-IR spectra (Spectrum Rx1, Perkin Elmer) were recorded in the range 4000–400 cm^−1a^ to a resolution of 4 cm^−1^.

### MTT Viability Assay

Vero cells from ATCC are used for the MTT assay. One hundred-microliter Vero cells at the concentration of 3 × 10^3^ cells/well were seeded in 96-well plates containing DMEM and incubated in 5% CO_2_ at 37 °C for 24 h. The medium was changed after 1 h and 100 μL of different concentrations (20,000, 10,000, 1000, 500, 250, and 100 μg/ml) of the 1.2 g progesterone-loaded nanofiber dissolved with PBS was added to the wells and incubated for 24 h at 37 °C in the CO_2_ incubator. One hundred microliter of MTT (5 mg/mL) was added to the wells containing cells and nanofibers of different concentrations. It was incubated at 37 °C for 4 h. The medium was then removed and 20 μL of DMSO was added to the wells. It was then shaken and incubated at 37 °C for 15 min and the absorbance was measured at 575 nm.

### In Vitro Release of Progesterone from Nanofiber

The in vitro release studies were performed at three different progesterone concentrations (1.2, 1.9, and 2.5 g) of nanofibers in a shaker at 37 °C. A weighed quantity of the fibers (20 mg) was suspended in PBS of pH 7.4. Then, it was kept in a shaker for seven days at 37 °C. The sample was withdrawn at regular one day intervals up to 7 days and replaced with the same volume of freshly prepared PBS pH 7.4. The withdrawn samples were used for OD measurement at 237 nm by a UV-visible spectrophotometer (Shimadzu, model UV-2601).

## Results and Discussion

In this, the electrospinning of zein nanofibers was mostly carried out by using ethanol system which resulted in ribbon-like fiber morphology due to the rapid mat formation of the fiber core because of the very fast evaporation of the solvent [15, 16].

### Characterization of Progesterone Loaded Zein Nanofiber

#### Scanning Electron Microscopy

**Fig. 1:**
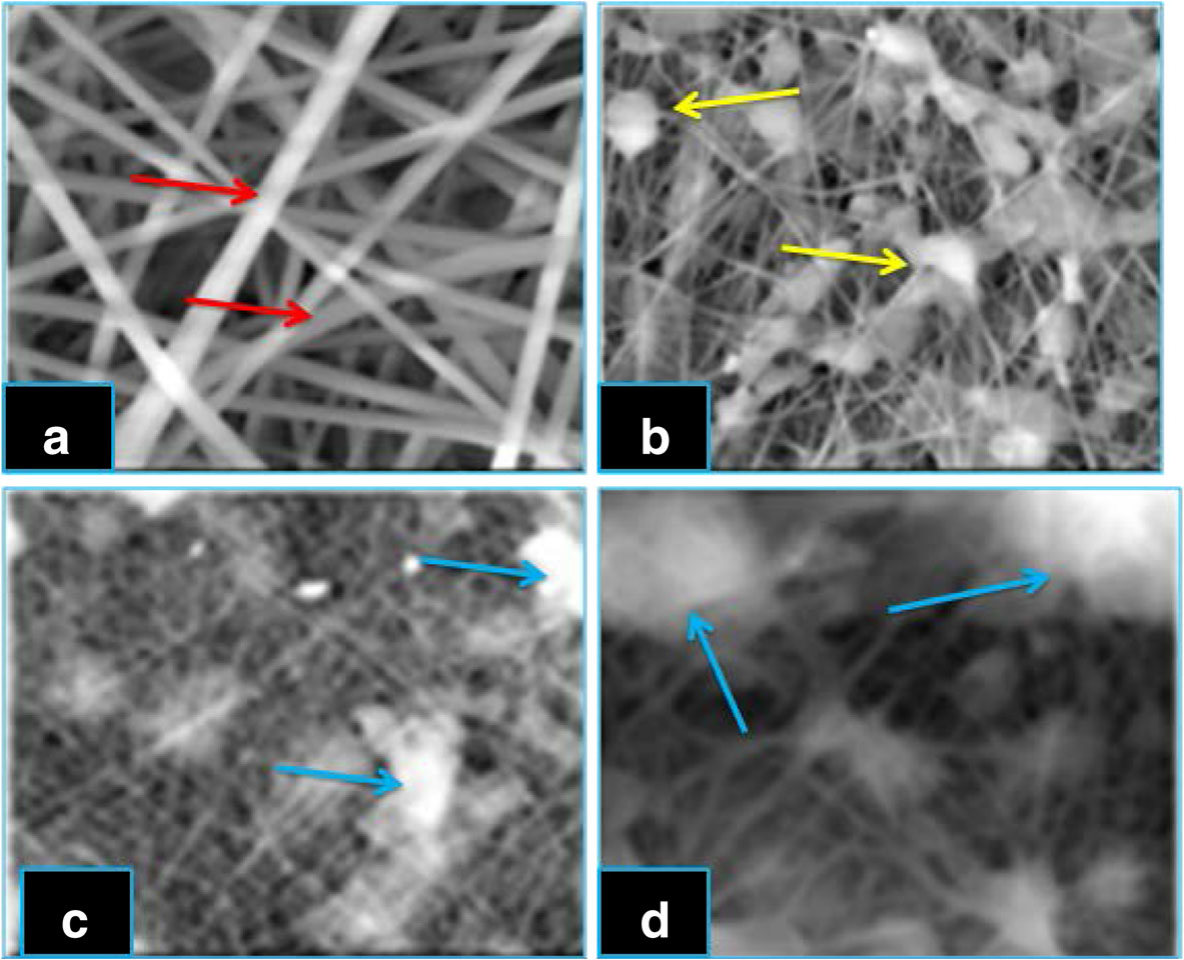
Scanning electron micrographs of zein nanofibers incorporated with various concentrations of progesterone, **a** without progesterone. *Red arrows* indicate the smooth and uniform surface morphology of nanofibers. Its shows not containing progesterone; **b** 1.2 g progesterone. *Yellow arrows* indicate the progesterone impregnated in the nanofibers; **c** 1.9 g progesterone, **d** 2.5 g progesterone. *Blue arrows* indicate the high concentration of progesterone disrupting the nanofiber structure

The fibers were spun from the same polymer solution and under the same spinning conditions with different concentrations of progesterone and were characterized by SEM. Zein nanofiber without progesterone had ribbon morphology with smooth surfaces and uniform structures (Fig. 1a) compared to the progesterone-impregnated nanofibers [17–19]. Progesterone was successfully impregnated on zein nanofiber and formed beads in the fiber mesh; it is clearly depicted in Fig. 1b. Among the three different concentrations, 1.2 g of progesterone-impregnated nanofiber entraps hormone both within their polymeric structures and within the minute interstitial spaces due to surface adsorption. When the concentration of progesterone increases, the nanofiber surface morphology was disrupted due to the increase in polymer solution viscosity creating difficulty in fiber formation; it is clearly depicted in Fig. 1c, d. However, 1.2 g of progesterone-interlocked fibers is suitable for sustained release of progesterone. The diameter of nanofiber without progesterone was around 170 nm. The average size of fiber diameter ranged from 180 ± 12 to 278 ± 16 nm for 1.2 g progesterone-impregnated zein nanofiber.

#### X-ray Diffraction Method

**Fig. 2:**
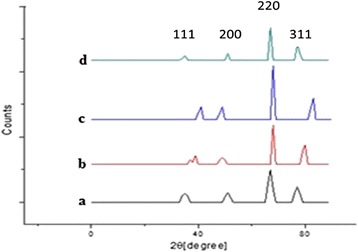
X-ray diffraction analysis of zein nanofibers incorporated with various concentrations of progesterone: *a* without progesterone, *b* 1.2 g progesterone, *c* 1.9 g progesterone, and *d* 2.5 g progesterone

The XRD patterns of electrospun zein nanofibers and different concentration of progesterone loaded nanofibers are shown in Fig. 2. Nanofibers without progesterone have shown two broad peaks having the maximum at 2θ = 64.3441 (1.44787 Å) and at 77.3335 (1.23391 Å) (Fig. 2a). Various concentrations of progesterone-loaded nanofiber show similar diffraction patterns (Fig. 2b–d). They are XRD patterns showing two broad halo diffraction patterns centered at 2θ = 63.2218 (1.25163 Å) and at 76.4672 (1.32872 Å) which are very similar to the zein nanofibers. Incorporation of progesterone at various concentrations did not affect the structural integrity of the nanofibers as no major shifts were seen. Formation of crystals is caused by the different extent of deformation of the polymer molecules during fiber formation by electrospinning [20–22]. The XRD pattern showed the character of zein and there is no new peak which can be confirmed that no chemical interaction between zein and progesterone in the formed nanofiber.

#### Differential Scanning Calorimetry

**Fig. 3:**
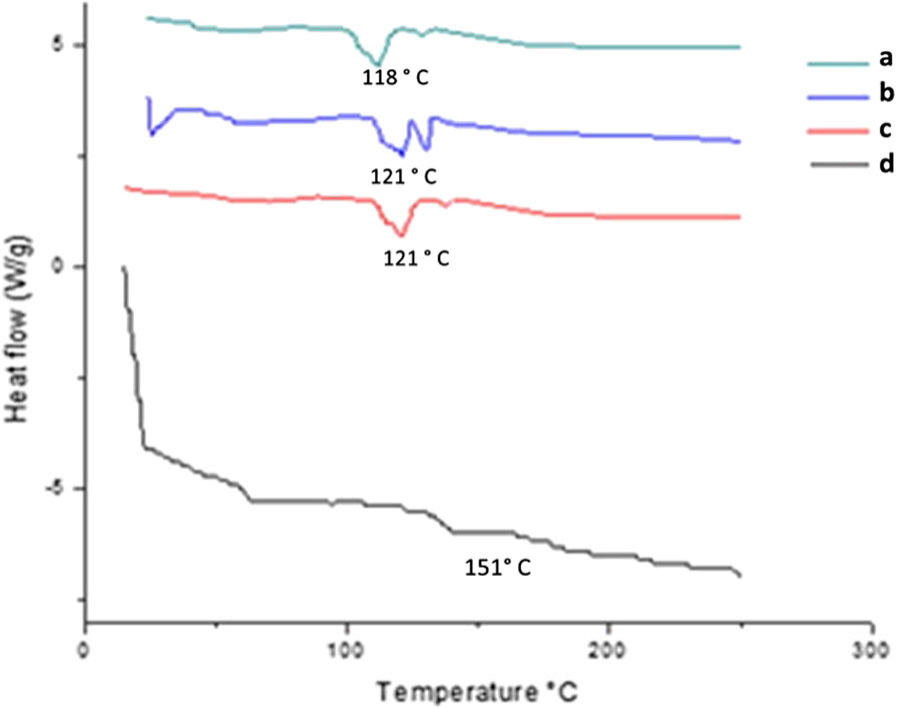
Differential scanning calorimetry results of nanofiber incorporated with various concentrations of progesterone: *a* without progesterone, *b* 1.2 g progesterone, *c* 1.9 g progesterone, and *d* 2.5 g progesterone

DSC was done on the electrospun nanofiber and progesterone incorporated nanofibers in order to determine the thermal behavior of the nanofibers. The glass transition temperature (Tg) of the different samples are presented in Fig. 3. The Tg of the nanofiber without progesterone was observed at around 151 °C, which is in close agreement with the Tg value reported in the literature for zein [23]. Different concentration of progesterone loaded zein fiber exhibited a sharp endothermic peak corresponding to the melting point of 121 °C for 1.2 g of progesterone loaded fiber, 121 °C for 1.9 g of progesterone loaded fiber, and 118 °C for 2.5 g progesterone loaded fiber (Fig. 3b–d). The addition of progesterone in the zein nanofibers caused a decrease in the Tg values which are possibly due to the plasticizing effect of the incorporated component [24–28]. Progesterone was integrated nicely with the zein molecules and displayed a plasticizing effect that increased the mobility of zein molecular chains.

#### FTIR

**Fig. 4:**
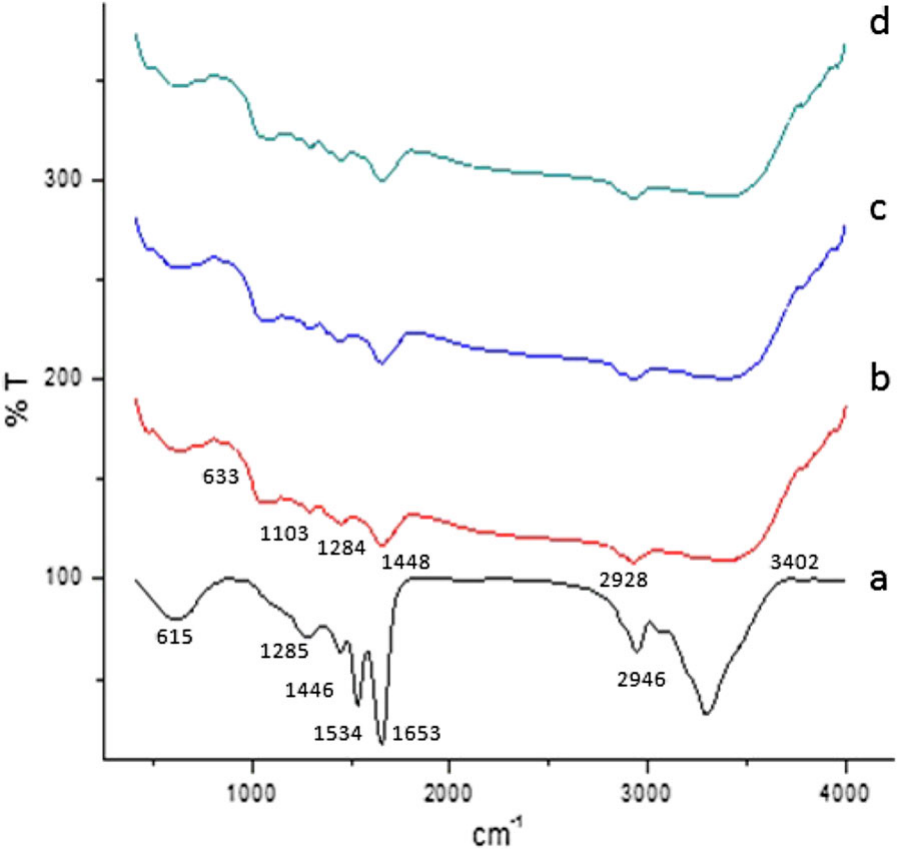
Fourier transform infrared spectroscopy results of zein nanofiber incorporated with various concentrations of progesterone: *a* without progesterone, *b* 1.2 g progesterone, *c* 1.9 g progesterone, and *d* 2.5 g progesterone

FTIR spectra of electrospun zein nanofibers and progesterone loaded nanofibers were shown in Fig. 4. The FTIR spectrum of zein nanofibers and progesterone loaded nanofibers were compared. The zein nanofiber spectrum showed strong bands at 615.94, 1285.59, 1446.75, 1534.47, 1653.63, 2358.82, 2946.97, and 3298.66 cm^−1^ (Fig. 4a). These bands correspond to the amide I and amide C–H deformation and bond vibration, trisubstituted aromatic ring, carboxylic acid, aromatic ring, C–O stretching, and acetylated lignin, respectively, for pure zein nanofibers. Different concentrations of progesterone-loaded nanofiber showed similar bands at 633.34, 1103.54, 1284.21, 1448.78, 1653.10, 2928.51, and 3402.89 cm^−1^ (Fig. 4b–d). These bands correspond to the amine N–H stretch, heterocyclic amine and NH stretch, alkenes, ketones, isocyanate aromatic functional group C–N stretch, and skeletal C–C vibration functional group [29]. Meanwhile, numerous peak sizes were reduced and some peaks totally disappeared from the spectra of progesterone loaded nanofibers compare to zein nanofiber spectra. These phenomena verify the speculation that hydrogen bonding had taken their roles in the formation of homogeneous composite fibers [30–32].

### MTT Assay

**Fig. 5:**
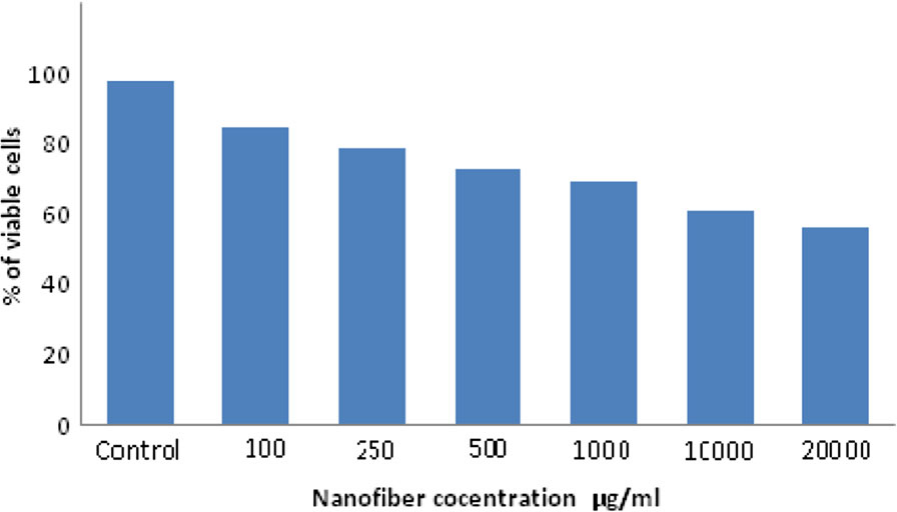
Graph of MTT assay after 24 h showing the rate of viability of Vero cells after exposure to different concentrations (20,000, 10,000, 1000, 500, 250, and 100 μg/ml) of the progesterone-loaded nanofiber

MTT assay was the most efficient, due to a less experimental error. The graph in Fig. 5 depicts the results of an MTT assay for different concentrations (20,000, 10,000, 1000, 500, 250, and 100 μg/mL) of the 1.2 g progesterone-loaded nanofiber added into Vero cells. Percent viability in the control sample and nearly 80% viability in the 100 μg/mL fiber added cells. In each high concentration, there was less reduction in the percentage of viability of cells. Sixty percent of cells are viable in the higher concentration of 20,000 μg/mL. Zein is one of the best-understood biomacromolecules and classified as Generally Recognized as Safe (GRAS) by the US Food and Drug Administration [33].

### In Vitro Release of Progesterone from Nanofiber

**Fig. 6:**
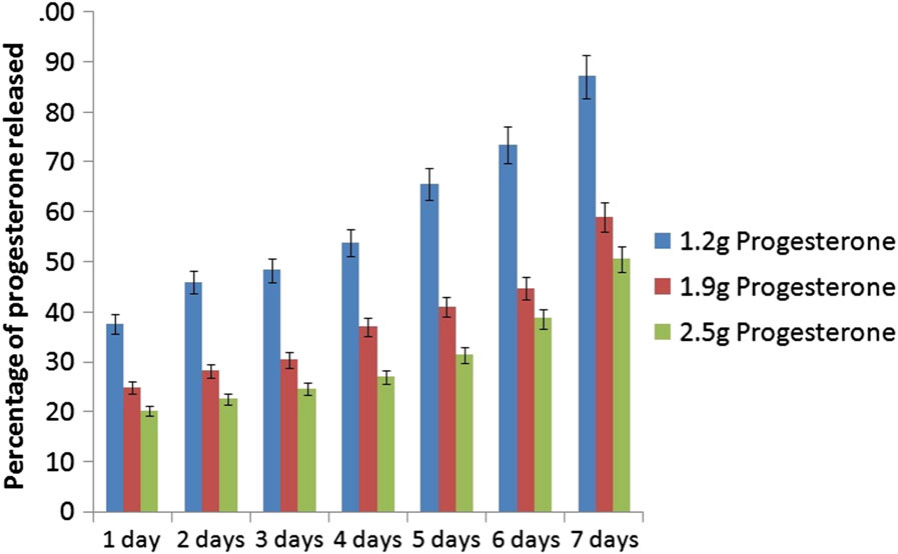
In vitro percentage progesterone release from zein nanofibers at different days post encapsulation

The percentage release of progesterone was also estimated for different concentrations of progesterone loaded and expressed in Fig. 6. Nanofibers loaded with 1.2 g of progesterone could release 87.28% of progesterone by 7 days and 50% release was achieved by 2.8 days. As the amount of progesterone loaded into nanofibers increased, the half life also increased correspondingly. This study confirms that 1.2 g progesterone loaded zein nanofibers can be potentially used in controlled delivering of progesterone, in livestock animals for estrus synchronization.

## Conclusions

The results of the current study confirm some miscibility of progesterone on hydrophobic biopolymers according to SEM, XRD, DSC, TGA, and FTIR. The electrospinning can be appropriately used to encapsulate active agents in biodegradable and biocompatible polymers, providing a hormone release sustainably. The increases in the concentration of progesterone affect the nanofiber size and morphology was confirmed by SEM. Progesterone at various concentrations did not affect the structural integrity of the nanofibers. Progesterone was found to have the effect of plasticizer when added to zein polymer. The electrospinning can be appropriated used to encapsulate active agents in biodegradable and biocompatible polymers, providing a hormone release sustainably. This study clearly indicated that 1.2 g progesterone-loaded zein nanofibers can be potentially used in controlled delivering of progesterone, in livestock animals for estrus synchronization.

## References

[1] Joanitti GA, Silva LP (2014). The emerging potential of by-products as platforms for drug delivery systems. Curr Drug Targets.

[2] Brettmann BK, Tsang S, Forward KM, Rutledge GC, Myerson AS, Trout AL (2012). Free surface electrospinning of fibers containing microparticles. Langmuir.

[3] Cao X, Deng WW, Fu M (2012). In vitro release and *in vitro in vivo* correlation for silybin meglumine incorporated into Hollow-type mesoporous silica nanoparticles. Int J Nanomedicine.

[4] Kayaci F, Uyar T (2012). Electrospun zein nanofibers incorporating cyclodextrins. Carbohydr Polym.

[5] Lai LF, Guo HX (2011). Preparation of new 5-fluorouracilloaded zein nanoparticles for liver targeting. Int J Pharm.

[6] Liu X, Lin T, Fang J, Yao G, Zhao H, Dodson M (2010). In vivo wound healing and antibacterial performances of electrospun nanofibre membranes. J Biomed Mater Res.

[7] Matabola KP, de Vries AR, Luyt AS, Kumar R (2011). Studies on single polymer composites of poly(methyl methacrylate) reinforced with electrospun nanofibers with a focus on their dynamic mechanical properties. Express Polymer Letters.

[8] Yu D, Zhu L, White K, Chris B (2009). Electrospun nanofiber-based drug delivery systems. Health.

[9] Yu M, Sun L, Li W, Lan Z, Li B, Tan T (2011). Investigation of structure and dissolution properties of a solid dispersion of lansoprazole in polyvinylpyrrolidone. J Mol Struct.

[10] Liu X, Lin T, Gao Y, Xu Z, Huang C, Yao G (2012). Antimicrobial electrospun nanofibers of cellulose acetate and polyester urethane composite for wound dressing. J Biomed Mater Res B Appl Biomater.

[11] Uyar T, Nur Y, Hacaloglu J, Besenbacher F (2009). Electrospinning of functional poly (methyl methacrylate) nanofibers containing cyclodextrin–menthol inclusion complexes. Nanotechnology.

[12] Uyar T, Hacaloglu J, Besenbacher J (2011). Electrospun polyethylene oxide (PEO) nanofibers containing cyclodextrin inclusion complex. J Nanosci Nanotechnol.

[13] Miyohi TK, Toyhara Minematsu H (2005). Preparation of ultrafine fibrous zein membranes via electrospinning. Polymer International.

[14] Nagy ZK, Balogh A, Vajna B, Farkas A, Patyi G, Kramarics A (2012). Comparison of electrospun and extruded Soluplus® -based solid dosage forms of improved dissolution. J Pharm Sci.

[15] Parris N, Cooke PH, Hicks KB (2005). Encapsulation of essential oils in zein nanospherical particles. J Agric Food Chem.

[16] Priya V, Kumar N, Sharma M, Pruthi V (2015). Biomedical applications of ferulic acid encapsulated electrospun nanofibers. Biotechnology Reports.

[17] Rathbone MJ, Macmillan KL, Bun CR, Burggraaf S (1997). Conceptual and commercially available intravaginal veterinary drug delivery systems. Adv Drug Deliv Rev.

[18] Rathbone JM, Bunt RC, Colin OR, Burggraaf S, Keith LM, Kim P (2002a) Development of an injection model poly(€-caprolactone) intravaginal insert for the delivery of progesterone to cattle. J Control Release 85:61–71 10.1016/s0168-3659(02)00272-9 12480312

[19] Oliveira EJ, Medeiros SE, Cardozo L, Voll F, Madureira HE, Capparelli HL, Benedito O (2010). Development of poly(lactic acid) nanostructured membranes for the controlled delivery of progesterone to livestock animals. Materials Science and Engineerinng C.

[20] Orawan S, Prasit P, Supaphol P (2011). Electrospun zein fibrous membranes using glyoxal as cross-linking agent: preparation, characterization and potential for use in biomedical applications. Chiang Mai J Sci.

[21] Rathbone JM, Bunt RC, Colin OR, Burggraaf S, Keith LM, Burke RC, Kim P (2002b) Reengineering of a commercially available bovine intravaginal insert (CIDR insert) containing progesterone. J Control Release 85:105–115 10.1016/s0168-3659(02)00288-2 12480316

[22] Souza SD (2014) A review of in vitro drug release test methods for nano-sized dosage forms. Advances in Pharmaceutics 1:1-12.

[23] Wallace SJ, Li J, Nation RL, Boyd BJ (2012). Drug release from nanomedicines: selection of appropriate encapsulation and release methodology. Drug Deliv Transl Res.

[24] Wang T, Kumar S (2006). Electrospinning of polyacrylonitrile nanofibers. J Appl Polym Sci.

[25] Torres-Giner S, Gimenez E, Lagaron J (2008). Characterization of the morphology and thermal properties of zein prolamine nanostructures obtained by electrospinning. Food Hydrocoll.

[26] Torres-Giner S, Ocio MJ, Lagaron JM (2009). Novel antimicrobial ultrathin structures of zein/chitosan blends obtained by electrospinning. Carbohydr Polym.

[27] Yu DG, Li X, Wang X, Chian W, Liao Y, Li Y (2013). Zero-order drug release cellulose acetate nanofibers prepared using coaxial electrospinning. Cellul.

[28] Zhang W, Chen M, Diao G (2011). Electrospinning [beta]-cyclodextrin/poly (vinyl alcohol) nanofibrous membrane for molecular capture. Carbohydr Polym.

[29] Wei K, Kim B, Kim B (2011). Fabrication and biocompatibility of electrospun silk biocomposites. Membranes.

[30] Xu L, Zheng R, Liu S, Song J, Chen J, Dong B (2012). NiO@ZnO heterostructured nanotubes: coelectrospinning fabrication, characterization, and highly enhanced gas sensing properties. Inorg Chem.

[31] Xu Y, Wang Y, Li XM (2014). Study on the release of fenofibrate nanosuspension in vitro and its correlation with in situ intestinal and in vivo absorption kinetics in rats. Drug Dev Ind Pharm.

[32] Brettmann BK, Cheng K, Myerson AS, Trout B (2013). Electrospun formulations containing crystalline active pharmaceutical ingredients. Pharm Res.

[33] Kumar R, Nagarwal RC, Dhanawat M, Pandit JK (2011). In-vitro and in-vivo study of indomethacin loaded gelatin nanoparticles. J Biomed Nanotechnol.

